# Factors Affecting Acceptance and Intention to Receive Pandemic Influenza A H1N1 Vaccine among Primary School Children: A Cross-Sectional Study in Birmingham, UK

**DOI:** 10.1155/2012/182565

**Published:** 2012-10-17

**Authors:** Michaela Janks, Sara Cooke, Aimee Odedra, Harkeet Kang, Michelle Bellman, Rachel E. Jordan

**Affiliations:** ^1^College of Medical and Dental Sciences, University of Birmingham, Edgbaston, Birmingham B15 2TT, UK; ^2^Unit of Public Health, Epidemiology and Biostatistics, University of Birmingham, Edgbaston, Birmingham B15 2TT, UK

## Abstract

UK pandemic influenza strategy focused on vaccination of high risk groups, although evidence shows that school-age children have the highest infection rates. Vaccination of children might be an additional strategy. We undertook a cross-sectional study amongst 149 parents of primary school children aged 4–7 years in Birmingham, UK to quantify intention to accept pandemic influenza vaccine and identify factors affecting uptake. Ninety-one (61.1%, 95% CI 52.8, 68.9) had or would accept vaccine for their child. The most common reasons for declining vaccine were concerns about safety (58.6% reported this), side effects (55.2%), or believing their child had already had swine flu (12.1%). Parents of nonwhite ethnicity (OR 2.4 (1.1, 5.0)) and with asthmatic children (OR 6.6 (1.4, 32.1)) were significantly more likely to accept pandemic vaccine, as were those whose children had ever received seasonal vaccine and those who believed swine flu to be a serious threat (OR 4.2 (1.9, 9.1)). Parents would be more likely to accept vaccination if they received a letter of invite, if the government strongly encouraged them, if it were administered at school, and if it were more thoroughly tested. Accurate media portrayal of safety of the vaccine during future pandemics will be essential.

## 1. Introduction 

The swine flu (H1N1) pandemic was confirmed on June 11th, 2009 by the World Health Organisation (WHO). The WHO declared the pandemic over by August 10th, 2010 [1], by which time 214 countries had reported laboratory confirmed cases, which included 18 449 deaths [2]. In contrast to seasonal influenza epidemics, in the 2009 H1N1 pandemic, younger age groups were disproportionately affected compared with older age groups [3]. A large proportion of older adults had preexisting natural immunity, probably due to HIN1 strains circulating in earlier decades [4]. Children under 5 years of age were most likely to be hospitalised if they contracted the H1N1 virus, and they also had high rates of admission to critical care with some fatalities [5]. 

In order to tackle the pandemic, plans worldwide were based on a vaccination programme and education [6]. In the UK, the vaccination programme officially started October 14th, 2009, with those in the “at risk” categories being offered the vaccination first. In December 2009, this was extended to children between the ages of six months and five years because of their increased level of risk [7]. However, sero-epidemiological studies based on the first wave of the pandemic showed that the rates of infection were actually the highest amongst school-aged children, where in London and the West Midlands (the areas of highest incidence), children aged 5–14 years had infection rates of approximately 42%, followed by the under 5s with infection rates of 21.3% [4]. This was approximately ten times the rate of people consulting with clinical influenza, highlighting the burden of mild and subclinical disease during the pandemic, and the importance of school children as vectors of transmission [4]. An additional strategy for future pandemics might be to extend vaccination to school-aged children to protect both themselves and the population via herd immunity. In some countries, this strategy is also used for seasonal influenza epidemics [8] as the evidence for the effectiveness of this strategy is beginning to accumulate.

There are few studies reporting likely uptake rates of this strategy among school-aged children, although a Mumsnet poll [9] indicated that 46% of parents of healthy under 5s would refuse (although it did not report on older age groups), and recorded uptake in England for the under 5s during the pandemic was only 23.6% [10]. In addition, most of the research about reasons for accepting/refusing influenza vaccine (either seasonal or pandemic) has been undertaken amongst healthcare workers [11–15] and more recently healthy adults [16–18]. Studies so far have revealed that there were low levels of anxiety towards the swine flu pandemic [19, 20]. This is believed to be due to early reports suggesting symptoms and prognosis of a similar severity to seasonal flu, encouraging the general population to consider themselves at low risk. Concerns about the safety of the swine flu vaccine and fear of adverse side effects have also been revealed to be important issues to address and this may be due to the misperception that the safety testing of such “fast-tracked” vaccines is insufficient, leaving a greater possibility of adverse health problems [20, 21]. These may have a particular effect when considering vaccinating children.

Given the threat of future pandemics, and also the high levels of H1N1 circulating in the subsequent 2010/11 influenza season, it is important to determine factors which might affect pandemic influenza vaccine uptake in young children in order to inform future vaccination policy decisions. We present a study undertaken during the 2009/10 influenza H1N1 pandemic among parents of primary school children to determine vaccine acceptance rates and factors affecting their decision to consent or not. 

## 2. Materials and Methods

### 2.1. Study Design

We undertook a cross-sectional survey among the parents of primary school children in Birmingham, UK to establish factors affecting uptake of (and intention to receive) pandemic influenza A H1N1 vaccination in the 2009/2010 season for their children.

### 2.2. Setting and Population

Parents of Key Stage 1 children (reception, years 1 and 2, i.e., ages 4–7 years inclusive) in the chosen schools who could read and understand English and were aged 18 years or over were included. 80 schools within the Birmingham Local Education Authority were randomly selected. These schools were contacted via e-mail to see if they would be interested in participating in the study. If, after 1 week, they had not responded (either favourably or not), they were called by telephone. The participating schools were sent questionnaires with cover letters, to be distributed to every child in Reception, Year 1 and Year 2. The schools were asked to give out the questionnaires, collect them back in within 1 week, and post them back to the investigators. 

### 2.3. Questionnaire

The questions were largely closed-ended and where possible were based on those used in similar studies previously carried out (the appendix), most importantly the paper by Zijtregtop et al. published recently in 2010, which used the Health Belief Model and the Behavioural Intention Model to derive their questions [21]. Piloting was undertaken among adults with children in the required age range. The questions included offer, acceptance, or intention to accept the vaccine for their child, demographic determinants, home circumstances, smoking status, health problems, previous influenza, and vaccination status. Seven out of the 25 questions additionally explored behavioural determinants, which were based on the Health Belief Model, including the following categories: perceived susceptibility, perceived severity, perceived barriers, and cues to action. Participants were also asked to provide reasons why they would or would not vaccinate their child. 

### 2.4. Outcome Measures

The main outcome was the intention to vaccinate their child or not. This was identified by asking *“Has your child been offered the swine flu vaccine this winter?”, “If yes, did they have the vaccination” and “If your child were offered it at some point in the future, would you vaccinate them against swine flu?”* A positive intention was defined for those participants answering “*Yes*” to either of the latter two questions. 

### 2.5. Statistical Analysis

Statistical analyses were undertaken in STATA 10. Simple descriptive statistics were used to describe the respondents and uptake rates. Multiple logistic regression analysis was undertaken to determine independent associations between specific factors and intention to receive the vaccine (providing odds ratios and 95% confidence intervals), adjusting for age, sex, ethnicity, smoking status, asthma status of child (model 1), and additionally for receipt of seasonal influenza vaccine (model 2). Outcomes with 5-point Likert scales were collapsed to 3 categories: agree/strongly agree, neither agree or disagree, and disagree/strongly disagree.

## 3. Results

### 3.1. Response Rate

Five schools agreed to take part in the study. 846 questionnaires were distributed to parents of children in Key Stage 1 in these schools, of which 149 were returned, giving a response rate of 17.6%. The highest response rate from a single school was 50% from school A and the lowest response rate was from school C (5.3%). 

### 3.2. Characteristics of Respondents

Of the 149 respondents, 118 (79.2%) were females (Table 1) and the most common age range of the study group was 36–40 years. The majority of the respondents lived with a partner (76.5%) and had never smoked (96/149 (64.4%)). The most commonly reported long-term illness among the children in the study was asthma (10.7% of the population). The largest ethnic group of our study population was white British or white other followed by people of Pakistani origin (28.2%). With regard to the children's vaccination history, 86.6% had had all routine vaccinations and 8.7% had received the seasonal influenza vaccine this year or previously. 

### 3.3. Knowledge and Opinions of Pandemic Influenza

111 (74.5%) respondents agreed/strongly agreed that they had a full understanding of the swine flu pandemic, while 13 (8.7%) felt that they did not (Table 2). 78 (52.4%) felt that the swine flu pandemic was a serious threat to society, although 23 (15.4%) disagreed. 27 (18.1%) felt that they were at high risk of getting swine flu. Nearly half of respondents used the television as their main source of information on current affairs (*n* = 74, 49.7%), followed by a further quarter (*n* = 38, 25.5%) who used the internet. 

### 3.4. Acceptance/Intention to Accept Pandemic Influenza Vaccine for Their Children

38 (25.5%) parents stated that they had been offered vaccine for their child (Table 3). Of these, 23 (60.5%) had accepted. Of the 111 not yet offered, 68 (61.3%) would agree for their child to receive the vaccine. In total, of all respondents, therefore, 91/149 (61.1%, 95% CI 52.8, 68.9) had a positive intention to vaccinate their child. In addition, 59/96 (61.5%) parents with other children under the age of 5 years would agree to them also receiving the vaccine in future. 

### 3.5. Reasons for and against Vaccination

Of those expressing positive intention to vaccinate, the main reasons included: “*worried about child catching swine flu*” (*n* = 21, 18.9%) and “*worried child would become severely ill if they caught swine flu*” (*n* = 11 (9.9%)) (Table 4). 

Of those expressing an intention to refuse the vaccine, the main reasons for not vaccinating their child included: *“worried about the safety of the vaccine”* (cited by *n* = 34/58, 58.6%) and *“worried about side effects”* (*n* = 32/58, 55.2%). 20.7% cited that they “*did not consider swine flu a threat*” as a reason for declining (Table 5). 

When asked which statements they agreed or strongly agreed with, 84 (56.4%) of parents stated they would be more likely to vaccinate their child if they received a letter inviting them to be vaccinated, 92 (61.7%) if the government strongly encouraged them, 72 (48.3%) if it were administered at school, and 98 (65.8%) if it were more thoroughly tested. 

### 3.6. Factors Affecting Intention to Accept Vaccine

Table 6 indicates factors affecting intention to accept pandemic influenza vaccine. On univariate analysis, the only statistically significant factors increasing acceptance were nonwhite ethnicity (OR 2.0 (95% CI 1.0, 3.9)), the child having asthma (OR 5.1 (1.1, 23.3)), and the child ever having seasonal influenza vaccine (OR 8.7 (1.1, 68.5)). In a model adjusted for age, sex, smoking status, ethnicity, and asthma in the child, non-white ethnicity (OR 2.4 (1.1, 5.0)) and having asthma (OR 6.6 (1.4, 32.1)) remained as significant factors. Trends for higher rates of acceptance were strengthened amongst current smokers and reduced amongst younger parents, but remained nonsignificant. The effect of receipt of seasonal influenza vaccine was in part explained by asthma in the child (model 2).

In an additional analysis adjusting for the factors above, respondents who agreed/strongly agreed that swine flu was a serious threat to society were significantly more likely to accept the vaccine (OR 4.2 (1.9, 9.1)). 

## 4. Discussion

### 4.1. Principal Findings

We investigated the factors that would influence acceptance of the swine influenza vaccination in primary school children. An important finding was that 61.1% of our study population were prepared to accept the swine flu vaccination if it were offered to their children (aged mainly over 5 years), and a similar proportion would also accept it for their younger children aged under 5 years. After multivariate analysis, three determinants were shown to have a significant association with positive intention to vaccinate. It was found that respondents of nonwhite (mainly Asian) origin were over twice as likely to accept the swine flu vaccine for their children than those who were of white ethnicity. Parents whose children had asthma were more than 6 times more likely to accept the vaccine than those who did not, and those who had received seasonal vaccine were also more likely (although some of this effect were likely to be explained by the child having asthma). It was also found that strong encouragement from the government, sending a letter to the parents and receiving the vaccination at school, would make the parents more likely to accept the vaccine. Concern about the safety of the vaccine and fear of side effects occurring were the main reasons given by participants who would not want their child vaccinated, and a large proportion of parents felt they would be more likely to consent to the vaccine if it were more thoroughly tested. More than 20% of parents cited a key reason for not agreeing to have their child vaccinated which was because they did not think swine flu was a serious threat to society. This is reflected by the finding that those who did think that swine flu was a serious threat were more than four times as likely to accept the vaccination for their children than those who did not.

### 4.2. Strengths and Limitations of the Study

Firstly, to our knowledge, this study is unique in that it examines factors affecting pandemic influenza vaccine uptake specifically among primary school children of this age. The questionnaires used were based around previously validated questionnaires used in similar studies [12]; therefore, interpretation of the questions by the respondents should not affect the results. However, although the sample was taken from a wide area across Birmingham with the schools initially being randomly selected to minimise selection biases, the low response rate among both schools and parents may affect generalisability and could also create a response bias. However, it is known that response rates to parental and other postal surveys are often low, and have generally been declining over time [22]. Our response rate varied from 5% to 50% depending on the school, which is consistent with other studies [22, 23]. 

School willingness to participate may be affected by different characteristics of the schools. These characteristics may also influence positive intention. The response rate was 17.6% which is low in comparison to another postal questionnaire in Birmingham [24]; however, it is not unexpected. It was also found that there was a greater response from School A which is located in a more affluent area (type 7 ACORN classification) [25] compared with School C (type 48) and School B (type 38). When dealing with health issues, it is a possibility that only those who are health conscious (and therefore likely to complete and send back the questionnaire) will respond. However, the converse may be true where people have strong feelings about vaccination; therefore, it is difficult to predict exactly how this might affect the uptake rates of vaccine and its determinants. 

Compared with the general population of Birmingham, in which 70.4% of the population classified themselves as white British/Irish/Other [26], only 46.3% of our study population also placed themselves in this category. The second largest ethnic group in our study population was found to be Pakistani (28.2%), and the third largest being Indian (12.1%). Therefore our study population does not reflect the overall ethnic structure of Birmingham. Different ethnicities vary in terms of positive intention rate, as shown in the multivariate analysis. This may partly explain the differences seen between acceptance in our study, and that reported in national statistics for the under 5s [10]. The national statistics may also include many people who were not offered the vaccine.

Furthermore, we note that our “intention to accept rates” is consistent with other studies as discussed below, which increases the confidence in our results.

Lastly, the small sample size in this study decreases its power, which decreases the probability that the study will be able to detect significant findings. Therefore, it is possible that some factors may have affected swine influenza vaccination uptake, but this study was unable to demonstrate this due to insufficient numbers.

### 4.3. Comparison with Other Studies

Although several studies have researched factors involved in uptake of the seasonal influenza vaccine, studies specific to the swine influenza pandemic have only recently been available [12, 13, 16–18, 27–29]. A recent systematic review (Nguyen et al.) of surveys conducted amongst the general public found that willingness to receive pandemic vaccine was very variable both within and across countries, ranging from 8% to 67%. A UK study before the swine flu vaccination campaign [16] indicated intention to receive vaccine among adults as 56.1%, and a further UK study [17] indicated that 60.8% of NHS workers and 74.6% of non-NHS workers were likely to have their child vaccinated, although a study in Turkey [28] indicated that only 33.9% healthcare workers would vaccinate their children. Our study is more consistent with the UK study. However, intention to vaccinate may overestimate eventual vaccination rates. In Rubin's 2011 telephone survey [18] 55.6% of NHS staff stated they would receive the vaccine although eventual acceptance rates for NHS staff recorded by the DH was 40%. For childhood vaccinations, the latest Health Protection Agency reports [30] indicate that the MMR vaccine has reached over 90% coverage. How this might translate into influenza vaccine coverage is not clear, although school-based influenza vaccination rates have reached over 70% in some places in the USA [31].

We found that the belief that swine flu is a threat to society positively influenced the uptake of vaccination. This accords with O'Reilly et al.'s study into factors affecting seasonal influenza uptake in health care workers, which found that beliefs about health were important determinants in the uptake of immunization [14]. We also found that parents of non-white ethnicity were more likely to consent to vaccination. In the UK random digit dialing, telephone surveys carried out during the early stages of the pandemic among 5175 adults [16] also showed that non-white ethnic groups were twice as likely to be likely to take up the swine flu vaccine (OR 2.0, (1.6, 2.6)), concurring with the results observed in our study. In addition, the cross-country systematic review of intention to receive vaccine also indicated that people of non-white ethnicity were significantly more likely to indicate willingness to receive pandemic vaccine [18]. A further cross-sectional telephone survey [19] of behaviour change in the UK in relation to the swine flu pandemic also revealed that the strongest predictor of behaviour change was ethnicity, where respondents from ethnic minorities were significantly more likely to undertake behaviours such as handwashing and avoiding large crowds than their white counterparts. The same study also showed that people with lower educational levels were more likely to change their behaviour, which is also indicated in our study, although not statistically significant. Other studies [21] have shown similar results. 

The main reasons for not receiving the vaccination in our study were found to be the fear of adverse reactions and worry about the safety of the vaccine. Similar findings were produced in 1976 North American studies which found that one of the principal reasons for declining vaccination was the worry that adverse reactions would occur. This may be due to the number of cases of Guillain-Barre syndrome occurring among recipients of swine flu vaccination during that time [32]. Other studies [11, 12, 28] also showed fear of side effects having a major role in declining the vaccine during this pandemic.

Low levels of anxiety towards the swine flu pandemic were shown in recent papers from the UK and Australia [19, 20]. Although 15.4% of our respondents seemed also to hold this view, more than 50% did believe it was a serious threat, and it was found to be an important factor affecting uptake. However, in the studies mentioned above, it is believed that the low anxiety levels were due to early reports suggesting that the symptoms and prognosis of swine influenza were similar to that of seasonal flu, and so the general population considered themselves at low risk. Only 18% in our study considered themselves at high risk of getting swine flu.

### 4.4. Implications

The findings of our study suggest that there is a relatively high positive intention to vaccinate if offered pandemic influenza vaccine. If this intention was followed through, then a vaccination programme among both primary school age children and under 5s would be worth carrying out in the event of future pandemics, particularly in pandemics when perceived threat was high. The way in which the swine influenza vaccination scheme is run would have a significant effect on the uptake of the vaccine. Strong government encouragement, sending a letter inviting children to be vaccinated or if the children were to receive the vaccination at school, was suggested to improve the chance that parents would accept swine flu vaccination. From these results, we would suggest that these methods are considered in the implementation of such a vaccination scheme in order to increase uptake rates. Clearly public views on the seriousness of the health risk and perceptions about the safety of the vaccine should be addressed with accurate media portrayal, particularly on television and the internet as these were found to be the main conduits of current affairs for our respondents. This season's experience from school-located vaccination programmes in the USA might also be useful for practical issues [33, 34]. In addition, further research would provide valuable insight into vaccination uptake, especially focussing on cues to action and the influence of ethnicity on vaccine acceptance. 

### 4.5. Conclusions

Understanding the factors involved in acceptance of the swine influenza vaccination is crucial for the effective implementation of future pandemic vaccination schemes. If swine influenza vaccination in children is accepted by the government as a worthwhile activity, then methods of maximising acceptance of the vaccine must be considered. Increasing uptake rates may be tackled in part by altering people's beliefs. Having some knowledge about who is likely to want the vaccination and why, and the reasons people may not want their children vaccinated, will allow health professionals to be prepared to answer patient's questions and concerns and the government to design their approach. Accurate media portrayal of the health risks of the pandemic and the safety of the vaccine is essential. Individual letters to parents from the government and administration of the vaccine in school would also appear to be important.

Given the high rates and complications of pandemic influenza in subsequent years, and the emerging interest internationally of a “herd immunity” approach, this research also has relevance for interim seasonal vaccination. 

## Figures and Tables

**Table 1:** Characteristics of respondents.

Characteristic	*N* (%)
Number of respondents	149
School	
A	46 (30.9%)
B	14 (9.4%)
C	5 (3.4%)
D	45 (30.2%)
E	39 (26.2%)
Females	118 (79.2%)
Age (years)	
<25	11 (7.4%)
26–30	30 (20.1%)
31–35	34 (22.8%)
36–40	42 (28.2%)
41+	32 (21.5%)
Number of children (mean) (SD)	2.4 (1.0)
Smoking status	
Never smoked	96 (64.4%)
Ex smoker	29 (19.5%)
Current smoker	24 (16.1%)
Ethnicity	
White British/other	69 (46.3%)
Mixed	10 (6.7%)
Indian	18 (12.1%)
Pakistani	42 (28.2%)
Other Asian	5 (3.4%)
Black	4 (2.7%)
Not stated	1 (0.7%)
Education of main earner	
No education completed	19 (12.8%)
Secondary	35 (23.5%)
College/vocational	47 (31.5%)
Degree or higher	48 (32.2%)
Long term illness	
None	101 (67.8%)
Child	14 (9.4%)
Parent/other member	22 (14.8%)
Combination	12 (8.1%)
Long term illness of child	
None	126 (84.6%)
Asthma	16 (10.7%)
Other	6 (4.7%)
Childhood routine vaccines	
Yes	129/149 (86.6)
No	15/149 (10.1)
Partially	5/149 (3.4)
Child ever received seasonal flu vaccine	
Yes	13/149 (8.7)
No	136/149 (91.3)

**Table 2:** Knowledge and attitudes to pandemic influenza.

	Agree/strongly agree	Neither agree or disagree	Disagree/strongly disagree
I have a full understanding of the swine flu pandemic (*n* (%))	111 (74.5%)	25 (16.8%)	13 (8.7%)
The swine flu pandemic is a serious threat to society (*n* (%))	78 (52.4%)	48 (32.2%)	23 (15.4%)
I feel I am at high risk of getting swine flu (*n* (%))	27 (18.1%)	64 (43.0%)	58 (38.9%)

**Table 3:** Pandemic swine flu vaccine acceptance among children.

	*N* (%)
Acceptance among children offered	23/38 (60.5%)
Intention to accept among children not offered	68/111 (61.3%)
Total positive intention to vaccinate child	91/149 (61.1%)
Future intention to vaccinate other children under 5 years of age	59/96 (61.5%)

**Table 4:** Main reasons for intention to accept pandemic vaccine.

Reason	*N* (%)
Worried about child catching swine flu	21 (18.9%)
Worried child would become severely ill if they caught swine flu	11 (9.9%)
Child has a long-term medical condition	8 (7.2%)
To prevent the child infecting other family members	8 (7.2%)
Followed advice from GP/school	8 (7.2%)
Children are more at risk than adults	7 (6.3%)
Prevent child having time off school	4 (3.6%)
Know others who have had swine flu	3 (2.7%)
Recommended vaccines should always be taken	1 (1.1%)

**Table 5:** Main reasons for refusing pandemic vaccine.

Reason	*N* (%)
Worried about the safety	34 (58.6%)
Worried about side effects	32 (55.2%)
Do not consider swine flu a threat	12 (20.7%)
Believe my child has already had swine flu	7 (12.1%)
Do not think the vaccine is effective	6 (10.3%)
Do not have time to go to the GP	3 (5.2%)
Against all vaccinations in general	3 (5.2%)

**Table 6:** Factors affecting intention to receive pandemic vaccine.

Factor	Positive intention *N* (%)	OR (95% CI)	Model 1 Adjusted OR^∗^(95% CI)	Model 2 Adjusted OR^†^(95% CI)
Age of parent (years)				
*>* *25 *	86 (62.3%)	1.0	1.0	1.0
< 25	5 (45.5%)	0.5 (0.1, 1.7)	0.3 (0.1, 1.3)	0.3 (0.1, 1.3)
Sex of parent				
Male	18 (58.1%)	1.0	1.0	1.0
Female	73 (61.9%)	1.2 (0.5, 2.6)	1.7 (0.7, 4.0)	1.7 (0.7, 4.1)
Smoking status of parent				
Never smoker	57 (59.4%)	1.0	1.0	1.0
Ex-smoker	17 (58.6%)	1.0 (0.4, 2.3)	1.4 (0.5, 3.7)	1.5 (0.5, 3.9)
Current smoker	17 (70.8%)	1.7 (0.6, 4.4)	2.3 (0.8, 6.5)	2.0 (0.7, 5.8)
Education of main earner				
Primary or less	14 (73.7%)	1.0	—	—
Secondary or higher	77 (59.2%)	0.5 (0.2, 1.5)	—	—
Ethnicity				
White	36 (52.2%)	1.0	1.0	1.0
Non-white	54 (68.4%)	2.0 (1.0, 3.9)	2.4 (1.1, 5.0)	2.5 (1.2, 5.5)
Parent vaccinated against swine flu				
No	74 (58.3%)	1.0	—	—
Yes	17 (77.3%)	2.4 (0.8, 7.0)	—	—
Child has asthma				
No	77 (57.9%)	1.0	1.0	1.0
Yes	14 (87.5%)	5.1 (1.1, 23.3)	6.6 (1.4, 32.1)	4.5 (0.8, 24.7)
Child ever received seasonal influenza vaccine				
No	79 (58.1%)	1.0	—	1.0
Yes	12 (92.3%)	8.7 (1.1, 68.5)	—	6.2 (0.7, 58.0)

^∗^Model adjusted for age, sex, smoking status, ethnicity, and asthma.

^†^Model adjusted for age, sex, smoking status, ethnicity, asthma, and receipt of seasonal influenza vaccine.

## Appendix

### Section A—Questions about the Parent/Guardian Completing This Form

1. Age of parent/guardian……
2. Sex (Please tick as appropriate)
  Male  □
  Female  □
3. How many children do you have living at home? (Number of children per age range)
  0–4 yrs……
  5–9 yrs……
  10–14 yrs……
  15+ yrs……
4. What is your current living situation?
  □ Living without partner
  □ Living without partner but with relatives
  □ Living with partner
  □ Living with partner and relatives
  □ Other
5. Smoking status:
  □ Current regular smoker
  □ Current occasional smoker
  □ Ex-smoker
  □ Never smoked
6. Highest level of education of the main earner:
  □ No education completed
  □ Secondary education completed (e.g., GCSEs or equivalent)
  □ College or Sixth form education completed (e.g., A-levels or equivalent)
  □ Vocational training
  □ Degree completed
  □ Masters completed
  □ PHD completed
7. Where do you usually get your information on current affairs from? (Please circle most appropriate)
  Broadsheet/Tabloid/TV/Internet/Magazine/None/Other
8. Do you or any member of your household have a long-term illness? (e.g., COPD, asthma, diabetes, heart disease, liver disease, kidney disease, MS, stroke) (Please circle all that apply)
  Child/Me/Other member/None
9. Please select one box per row/statement
10. I have a full understanding of the swine flu pandemic
  □ Strongly Agree
  □ Agree
  □ Neither Agree or Disagree
  □ Disagree
  □ Strongly Disagree
11. The swine flu pandemic is a serious threat to society
  □ Strongly Agree
  □ Agree
  □ Neither Agree or Disagree
  □ Disagree
  □ Strongly Disagree
12. I feel I am at a high risk of getting swine flu
  □ Strongly Agree
  □ Agree
  □ Neither Agree or Disagree
  □ Disagree
  □ Strongly Disagree
13. Do you know anyone that has had swine flu (including yourself)?
  Yes/No
14. Did you have the swine flu vaccination?
  Yes/No

### Section B—Questions about your Child

If you have more than one child, please answer for the child in year 1/2 that brought this questionnaire home

(14) Does your child have a long-term illness?
  Asthma/Other respiratory illness/Other long-term illness/None
(15) Do you consider your child to have a disability?
  Yes/No
(16) Has your child had all the vaccinations recommended by your GP (including MMR)?
  Yes/Partially/No
(17) Has your child ever had the seasonal flu vaccine?
  Yes/No
(18) Has your child been offered the swine flu vaccine this winter?
  Yes/No (If no, go to question (20))
(19) If yes, did they have the vaccination?
  Yes/No (If no, go to question (21))
(20) If your child were offered it at some point in the future, would you vaccinate them against swine flu?
  Yes/No (If no, go to question (21))

If you've answered yes to either question (19) or (20), please describe the factors contributing to this decision

(21) If no, what factors contributed to this decision? (Please tick any that apply)
  □ Do not think the vaccine is effective
  □ Worry about side effects of vaccine
  □ Worry about the safety of the vaccine
  □ Can't find the time to go to the GP
  □ Do not consider swine flu as a threat
  □ I believe my child has already had swine flu
  □ I am against vaccinations in general
  □ Other (please specify)
(22) I would be more likely to vaccinate my child if:
  (Please tick one option per row/statement)
  I received a letter inviting them to be vaccinated
    □ Strongly Agree
    □ Agree
    □ Neither Agree or Disagree
    □ Disagree
    □ Strongly Disagree
  The government strongly encouraged children of my child's age to have it done
    □ Strongly Agree
    □ Agree
    □ Neither Agree or Disagree
    □ Disagree
    □ Strongly Disagree
  The vaccine was given at my child's school
    □ Strongly Agree
    □ Agree
    □ Neither Agree or Disagree
    □ Disagree
    □ Strongly Disagree
  The vaccine was tested more thoroughly
    □ Strongly Agree
    □ Agree
    □ Neither Agree or Disagree
    □ Disagree
    □ Strongly Disagree
(23) Regarding any of your OTHER children up to the age of 5 years—if offered, would you consent to their having swine flu vaccination?
  Yes/No/do not have any others of this age-group

### Section C

(24) What is your ethnic origin?
*White*: British/Irish/Other White
*Mixed*: White and Black Caribbean/White and Black African/White and Asian/Other Mixed
*Asian or Asian British*: Indian/Pakistani/Bangladeshi/Other Asian
*Black or Black British*: Black Caribbean/Black African/Other Black
*Chinese or other*: Chinese/Other Ethnicity
(25) How long have you lived in the UK?

Thank you for taking the time to complete our questionnaire!
